# Time-restricted eating to address persistent cancer-related fatigue among cancer survivors: A randomized controlled trial

**DOI:** 10.21203/rs.3.rs-5530166/v1

**Published:** 2024-12-25

**Authors:** Amber S. Kleckner, Carin L. Clingan, Shari M. Youngblood, Ian R. Kleckner, Lauren Quick, Rebecca D. Elrod, Shijun Zhu, Emily N. C. Manoogian, Satchidananda Panda, Ashraf Z. Badros, Ashkan Emadi

**Affiliations:** University of Maryland School of Nursing; University of Maryland School of Nursing; University of Maryland School of Nursing; University of Maryland School of Nursing; University of Maryland School of Nursing; University of Maryland School of Nursing; University of Maryland School of Nursing; The Salk Institute for Biological Sciences; The Salk Institute for Biological Sciences; University of Maryland School of Medicine; University of Maryland School of Medicine

## Abstract

**Purpose:**

Time-restricted eating (TRE) helps regulate rest-activity rhythms, blood glucose, and other diurnally regulated energetics processes, which may have implications for persistent fatigue. In a randomized controlled trial, we tested the effects of TRE vs. control on fatigue in cancer survivorship.

**Methods:**

Adult cancer survivors were recruited who were 2 months to 2 years post-treatment and reported moderate to severe fatigue. Participants were randomized 1:1, TRE:control and all received individualized nutrition counseling. The TRE group self-selected a 10-hour eating window for 12 weeks. At baseline, week 6, and week 12, participants were asked to log eating instances, complete the Functional Assessment of Chronic Illness Therapy-Fatigue questionnaire (FACIT-F, higher score=less fatigue), and wear an actigraph and continuous glucose monitor.

**Results:**

Thirty participants completed baseline assessments and were randomized (77% female, 53% Black/African American, 43% White, 7% Hispanic; 54.1±14.7 years old; 87% with blood cancer); 25 completed 12-week assessments. TRE led to a meaningful reduction in fatigue at week 12 controlling for baseline levels (change in FACIT-F fatigue subscale=0.0±5.4 for control, 4.1±5.7 for TRE, *p*=0.11, effect size [ES]=0.70; clinically meaningful threshold=3.0 points). Glucose parameters (e.g., average interstitial glucose, average fasting glucose) tended to be lower and rest-activity rhythms tended to indicate more regularity for those in the TRE vs. control group at weeks 6 and 12, though differences were not statistically significant (*p*>0.19).

**Conclusions:**

A 12-week, nutritionist-led TRE program led to less fatigue than control. Continued study of TRE patterns are warranted to optimize this eating pattern and address persistent cancer-related fatigue.

## Introduction

Cancer-related fatigue is one of the most prevalent and distressing symptoms experienced by cancer patients, with approximately 42% of cancer survivors reporting persistent fatigue more than 3 months post-treatment [1]. Unlike the tiredness from typical daily activities, cancer-related fatigue is more severe, persists despite rest, and interferes with daily functioning and quality of life [3]. It can persist for months or even years after treatment, contributing to a diminished capacity for physical and cognitive activities, emotional distress, and reduced social interactions [4]. Given its substantial negative impact on the lives of cancer survivors, effective strategies to mitigate cancer-related fatigue are of critical importance [5].

The factors underlying cancer-related fatigue are multifactorial and complex [38], and nutritional interventions may be able to address upstream factors [15]. Specifically, circadian rhythms are sometimes disrupted in cancer patients [35, 37] and may precipitate and/or perpetuate fatigue [14, 39]. Time-restricted eating (TRE), also known as prolonged nighttime fasting, has recently garnered attention for its potential to entrain and sustain circadian rhythm [16, 18]. TRE is a dietary strategy that involves consuming all meals within a specific time window of less than 12 hours per day. By aligning food intake with the body’s natural biological clock, TRE may enhance sleep efficiency at night, allow the body to more reliably and accurately predict energy availability and expenditure, and promote increased energy levels during the day [8].

Scientific literature to support TRE as a strategy to address cancer-related fatigue is emerging, as TRE can positively influence metabolic and circadian health [17, 42]. Animal studies have demonstrated that TRE can improve circadian rhythm robustness, which is linked to better sleep and reduced fatigue [7, 13]. Additionally, preliminary single-arm human studies suggest that TRE is feasible among cancer survivors and may help alleviate fatigue [17, 19, 20, 30]. However, randomized controlled trials are needed to evaluate TRE vs. a control group to estimate the specific effects of TRE on cancer-related fatigue.

Herein, we conducted a small randomized controlled trial to test the feasibility of a randomized TRE study as well as test the hypothesis that TRE vs. a time- and attention-matched control intervention (nutrition counseling without a time component) can reduce cancer-related fatigue.

## Methods

### 2.1 Study design

The Fatigue REDuction After cancer (FREDA) trial was a pilot randomized controlled trial conducted through University of Maryland Medical System from January 2023 to June 2024 (NCT05256888, registered February 2022). The research protocol was reviewed and approved by the University of Maryland Institutional Review Board (IRB; HP-00099067) and conducted in accordance with the Declaration of Helsinki. The primary aims were feasibility and to test the effects of TRE vs. an unrestricted eating group on cancer-related fatigue. Secondary aims were to evaluate the effects of TRE on glucose regulation and rest-activity rhythm, as described below. We targeted enrollment survivors of hematological malignancies given the high prevalence of persistent fatigue (58% [1]) and high interference of fatigue with daily activities in this population [40, 41].

### 2.2 Eligibility criteria

Inclusion criteria (Participants must…): be 2 months to 2 years post-treatment with chemotherapy, surgery, and/or radiation for hematologic malignancies or solid tumors; have a baseline level of fatigue, as assessed by responding 4 or higher to the question, “In the last week, how bad was your worst fatigue on a scale from 0–10?”; be at least 18 years old; be able to communicate in English; and have access to a smartphone or mobile device. Exclusion criteria (Participants must not…): already eat all their food within a window that is 10 hours or shorter most (six of seven) days per week; have a body mass index ≤18.5 kg/m^2^; have surgery planned during the study duration, and have any contraindications to the proposed intervention (e.g., insulin-dependent diabetes, enteral or parenteral nutrition, recent history of an eating disorder). Individuals were eligible if they were on maintenance therapy with non-conventional chemotherapy (e.g. targeted therapy).

### 2.3 Procedures

Participants consented to the study either on paper or electronically via Research Electronic Data Capture (REDCap) tools hosted at University of Maryland Baltimore [12, 22]. After consent, participants were in the study for approximately 14 weeks. During the baseline week (7 days), participants were asked to log their food; wear an actigraphy watch (MotionWatch8, CamNTech, Boerne, TX, USA); wear a continuous glucose monitor (FreeStyle Libre, Abbott Nutrition, Chicago, IL, USA); and complete questionnaires (described below). After baseline assessments, participants were randomized 1:1 to the TRE group (intervention) or the unrestricted eating group (control). The randomization table was generated in block sizes of 2 and 4 by the statistician (SZ) and concealed from the other study team members using REDCap; there was no stratification. All participants met with a licensed dietitian nutritionist (SMY) for individualized nutrition counseling. Those assigned to the TRE group were asked to meet recommendations within a self-selected 10-hour eating window while those in the control group were not given temporal restrictions. Participants were asked to follow the recommendations for 12 weeks. A coordinator or the nutritionist checked in with each participant at least every 2 weeks. The same data collection protocol was conducted at week 6 and week 12. Data collection and meetings were conducted completely remotely, and equipment was mailed to and from participants’ homes.

### 2.4 The control arm

Participants met with the nutritionist after randomization for individualized counseling. Meetings were via phone or Zoom and lasted approximately 30–60 minutes. Participants discussed eating habits and set goals based on their individual needs—macronutrients, food groups, maximum servings of nutrient-poor selections, food preferences, culinary self-efficacy, etc. Participants were not asked to change the timing of their food intake.

### 2.5 Time-restricted eating (TRE) intervention

Those randomized to TRE selected a 10-hour eating window based on schedule and preferences. We encouraged the eating window to be during the day and end several hours before bedtime. Aside from water, which was always allowed, only unsweetened tea and black coffee were allowed before the eating window; calorie-free foods and beverages such as chewing gum and diet soda were not allowed outside the eating window. Participants met with the same nutritionist as those in the control group for individualized nutrition counseling, and goals discussed during the counseling session were asked to be met within the eating window. To match expectancy, both groups were told that nutritional programs can help with fatigue, and we are not sure the restricted eating window will help or not.

### 2.6 Outcomes

#### 2.6.1 Adherence

Participants were declared adherent to TRE if their average eating window was ≤10 hours. Eating windows were assessed using the myCircadianClock smartphone application [26]; paper-based logs were permittable as an alternative to the app. We calculated the eating window from the first and last calorie entered every day at baseline, week 6, and week 12, then averaged the eating window across the 7 days. If a participant logged only one eating instance within a day or if the eating window was <4 hours, we declared that day to have missing data.

#### 2.6.2 Fatigue

Questionnaires were administered at baseline, week 6, and week 12. Fatigue was assessed using the Functional Assessment of Chronic Illness Therapy-Fatigue (FACIT-F) [6] and the Brief Fatigue Inventory (BFI) [28]. The FACIT-F is a 40-item, validated patient-reported fatigue measure that is comprised of five subscales: physical well-being, social well-being, emotional well-being, functional well-being, and fatigue [6]. Participants respond how true various statements were over the last seven days such as “I feel fatigued” and “I have to limit my social activities because I am tired” with five response choices ranging from 0, “Not at all,” to 4, “Very much.” Scoring, which involves reversing some items, yields five subscale scores and a total score. A higher score indicates higher well-being/quality of life or less fatigue. The BFI is a 10-item fatigue questionnaire that is also validated and commonly used in oncology [28]. It captures fatigue *now* as well as the *usual* and *worst* fatigue in the last 24 hours from 0, “No fatigue,” to 10, “As bad as you can imagine.” It also includes six single-item questions regarding how fatigue has interfered with general activity, mood, etc., from 0, “Does not interfere” to 10, “Completely interferes.” The average of all 10 items yields a global fatigue score with a higher score indicating worse fatigue. Cronbach alpha reliability ranges from 0.82 to 0.97 [28].

#### 2.6.3. Body weight

Participants were given a scale (Weight Watchers, Conair Corp., Stamford, CT) and were asked to record body weight weekly, soon after waking with minimal clothing.

#### 2.6.4 Glucose monitoring

Participants were provided a continuous glucose monitor (Freestyle Libre) to wear on the back of their upper arm for 7 days at baseline, 6 weeks, and 12 weeks. Interstitial glucose was recorded every 15 minutes, and data were extracted using LibreView (Abbott). Data were manually checked for quality and any traces that showed a large (>50 mg/dl) stepwise shift in average values or unreasonable values (>350 mg/dl) were removed. Average, daily maximums and minimums, fasting glucose, and coefficient of variation were extracted from glucose traces by a researcher blinded to treatment groups (LQ) [27].

#### 2.6.5 Actigraphy

Participants were asked to wear a MotionWatch8 (CamNTech) on their wrist of choice for 24 hours per day for 7 days at baseline, 6 weeks, and 12 weeks. The actigraphy watch measured triaxial activity “counts” in 15-second epochs. Using MotionWare software (CamNTech), periods during which the watch was worn were manually selected by an assessor blinded to treatment group (RDE); parametric measures (cosine peak, amplitude, midline estimating statistic of rhythm [MESOR]) and non-parametric measures (interdaily stability, intradaily variability, activity in the 5 consecutive hours with the least activity, activity in the 10 consecutive hours with the most activity, relative amplitude) of activity were extracted [34].

### 2.7 Statistical analysis

Differences between groups (randomized vs. not and TRE vs. control) at baseline were assessed using a *t*-test for continuous variables, a χ^2^ test for categorical variables. Linear mixed models were used to assess the intervention effects on fatigue and all mechanistic measures. We stated *a priori* that we would use an analysis of covariance (ANCOVA) to assess the effects of group at week 6 and week 12 but, due to the large amount of missing data, we decided that a mixed model would be more appropriate. Thus, results of both models are reported for FACIT-F fatigue outcomes. Mixed effect models were constructed with group, time (continuous), and group×time as fixed effects and participant as a random effect. Effect sizes were calculated as Cohen’s d (Δ_TRE_ –Δ_Control_)/SD_pooled_, where Δ is the change from baseline to week 6 or week 12 and SD_pooled_ is the standard deviation from all participants at baseline [23]. A two-sided *p*<0.05 was used to assess between-group differences in demographics and clinical characteristics, and a probability of *p*<0.15 was declared *a priori* to be considered meaningful for informing future research in regard to effects of time and group on outcomes [36]. No interim analyses were planned or performed.

## Results

**Figure 1:**
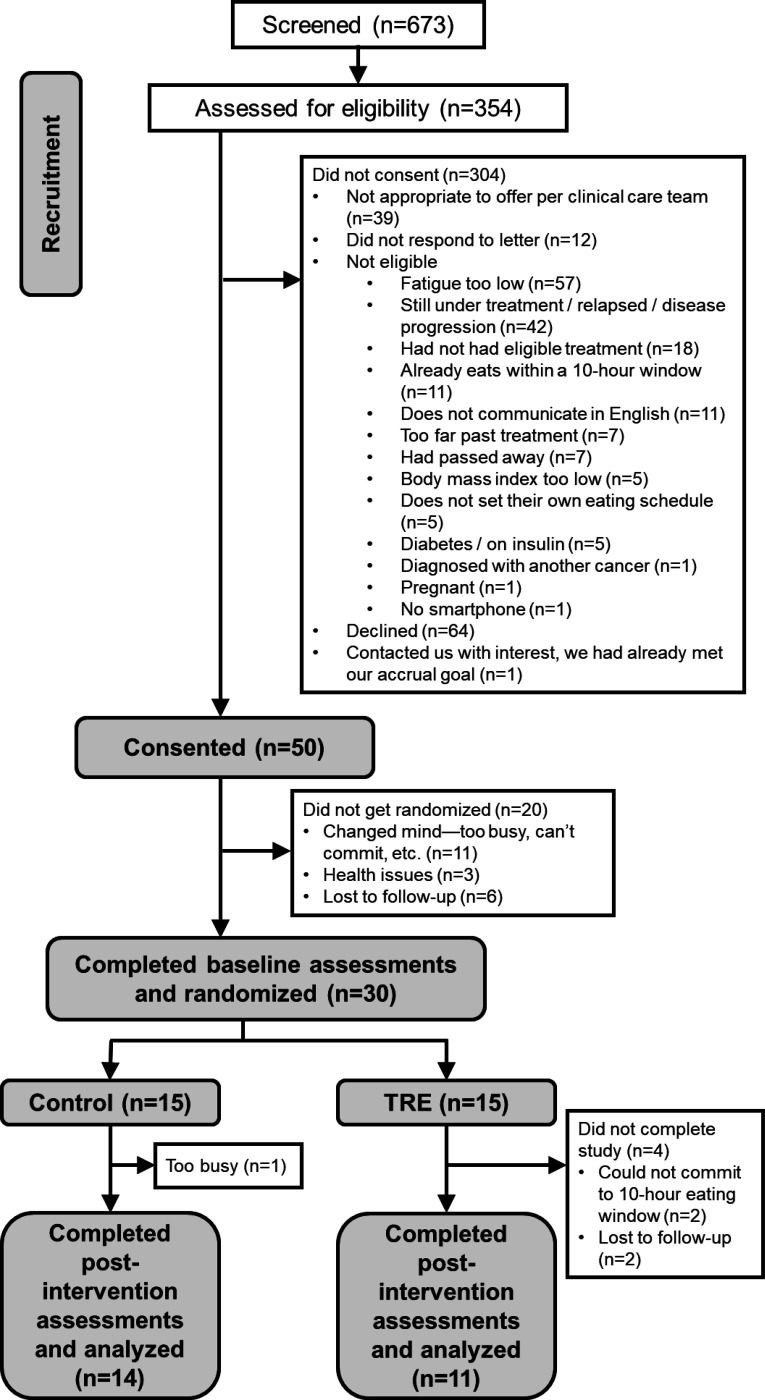
Consolidated Standards of Reporting Trials (CONSORT) diagram

Fifty participants were enrolled between January 2023 and February 2024 and 30 were randomized (Fig. 1; Table 1). Those who withdrew or were lost to follow-up before randomization tended to be older (*p*=0.0048) and have less education than those who completed the study (*p*=0.0098). Those who withdrew vs. were retained had similar distributions for race, sex, living situation, employment, cancer type, comorbidity index, and baseline FACIT-F fatigue subscale scores (*p*>0.05). Those randomized to the TRE vs. control group had similar demographics and clinical characteristics (all *p*>0.085).

To monitor adherence, eating windows were calculated from food logs entered into myCircadianClock [26] (*n*=21) or a paper-based log (*n*=2). However, only 23/50 (46%) participants provided any data at baseline, 19/30 (63%) provided data at week 6, and 13/30 (43%) provided data at week 12 (Suppl. Fig. 1). The average eating window reported at baseline was 10.5±1.8 hours. Of those who logged eating instances, 9/10 of respondents in the TRE group consumed food within an eating window ≤10 hours at week 6 (8.9±0.6 hours) and 4/4 at week 12 (8.6±0.9 hours, Suppl. Table 1).

**Figure 2:**
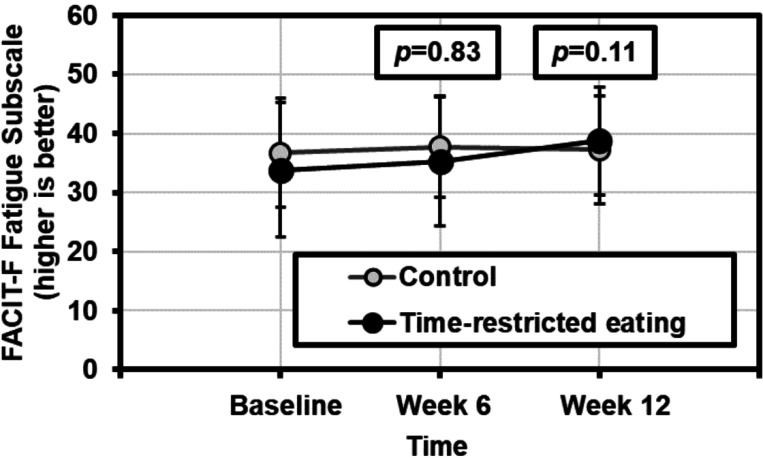
Fatigue, as measured using the Functional Assessment of Chronic Illness Therapy-Fatigue (FACIT-F) fatigue subscale, over time for those in the time-restricted eating vs. control groups. P-values are a result of Analysis of Covariance (ANCOVA) models constructed at week 6 (effect size=0.27) and at week 12 (effect size=0.70) with baseline values as covariates.

On average, fatigue improved over time as measured using the FACIT-F fatigue subscale (mixed model effect of time, β±SE=0.17±0.09, *p*=0.059; Table 2). Further, there was a between-group difference in FACIT-F fatigue scores favoring the TRE group (group×time β±SE= −0.17±0.09, *p*=0.069). At week 6, the magnitude of FACIT-F fatigue subscale scores was higher (less fatigue) in the TRE group controlling for baseline values, but the between-group difference did not meet statistical significance (ANCOVA, change from baseline to week 6=2.5±4.5 for the TRE group vs. 0.9±7.7 in the control group, *p*=0.83, effect size [ES]=0.27; Fig. 2). At week 12, differences between groups were larger (4.1±5.7 for TRE vs. 0.0±5.5, *p*=0.11, ES=0.70). The improvements in fatigue in the TRE group exceeded the minimal clinically important difference (3.0 points [29]). Trends were similar as measured by the global BFI score and other subscales of the FACIT-F and BFI in that fatigue improved slightly from baseline to week 12 and the TRE group experienced greater benefits with small-moderate effect sizes on average (Table 2).

Among those who provided body weight data (*n*=15 in the control group and *n*=10 in the TRE group), those in the control group tended to lose a small but statistically significant amount of weight over time (β±SE= −0.024±0.009 pounds/day, *p*=0.013) and those in the TRE group gained a small but statistically significant amount of weight over time (β±SE=0.031±0.015 pounds/day, *p*=0.038). The between-group difference was statistically significant (β±SE= −0.027±0.008, *p*=0.002).

The effects of TRE vs. control on glucose regulation were measured using continuous glucose monitoring (Suppl. Fig. 2). Glucose monitoring was feasible for about half of our active participants. Glucose parameters tended to be lower at week 6 and week 12 for those in the TRE group, though no measures met statistical significance (*p*>0.25; Suppl. Fig. 3, Suppl. Table 2). For example, average daily minimum was lower for those in the TRE group compared to the control group at week 6 (change from baseline= 4.4±16.0 mg/dl in the control group and −13.0±11.6 in the TRE group) and at week 12 (change from baseline= 0.7±17.7 mg/dl in the control group and −6.3±18.1 mg/dl in the TRE group, mixed model group×time estimate ± SE = 0.29±0.25, *p*=0.258).

We collected actigraphy data to quantify the strength of participants’ rest-activity rhythm, or a person’s regular daily 24-hour pattern of being active and resting. Stronger rest-activity rhythms are reflected by high activity during the day and low activity at night (i.e., high fitted cosine amplitude) and regular movement and rest patterns each day (i.e., interdaily stability; Table 3). Actigraphy data were available from 33 participants and 24 contributed data for at least two time points (Suppl. Fig. 4). Three participants removed their actigraphs at night, precluding our ability to calculate parametric rest-activity measures. The estimate for group×time interaction in mixed models for all rest-activity parameters suggested that TRE led to higher regulation of rest-activity rhythms, though this term did not reach statistical significance for any model (*p*>0.19 for all parameters that reflect the degree of rhythm entrainment, Table 3, Suppl. Fig. 5).

In regard to safety, there were two grade 4 adverse events and none of these adverse events were attributed to the intervention or study procedures. Two hospitalizations occurred in the control group—one for shortness of breath and one for pneumonia. There was one grade 3 adverse event: approximately 2 weeks of nausea and diarrhea that led to significant weight loss in the TRE group.

## Discussion

This is one of the first randomized controlled trials to test the effects of TRE on cancer-related fatigue. Throughout the 12-week trial, fatigue tended to be stable in the control group and tended to gradually decrease in the TRE group to a clinically meaningful degree (> 3 points), yielding a moderate effect size of 0.70 at week 12 as measured using the FACIT-F fatigue subscale (Table 2). Collection of continuous glucose monitor data and actigraphy was feasible for more than half of our participants, though some could not apply the glucose monitor on their own, chose not to wear the device(s), or did not return the device(s) to our lab. While participants did not necessarily have impairments in glucose regulation, TRE led to slight decreases in average and fasting interstitial glucose concentrations, which tends to reflect healthier levels. Similarly, actigraphy data suggest that TRE may help increase the robustness of rest-activity rhythms, but this study was not powered adequately to resolve group-level differences. Throughout the course of the study, we increased our engagement strategies and only two of our last 14 consents withdrew before randomization. Thus, after troubleshooting our recruitment and retention strategies, recruitment to the TRE study was feasible and participants were retained. These data support continued investigation into TRE to better understand how to leverage dietary patterns to regulate circadian rhythms and energy metabolism to address fatigue.

These results build on promising single-arm interventional trials that support the use of TRE to regulate circadian rhythms and address cancer-related fatigue [16, 18]. For example, in a single-arm TRE study, a cohort of survivors of mixed cancer types (*n* = 36) reported less fatigue after two weeks (mean pre-post improvement of 5.3 points on the FACIT-F fatigue subscale, effect size = 0.55) [20]. In addition, breast cancer survivors (*n* = 40) saw a pre-post improvement in fatigue after 12 weeks of TRE (median change of 1.0 points on the FACIT-F fatigue subscale) [30]. In populations other than cancer populations, studies tend to report increased energy levels that are sustained for up to one year or no increases in fatigue (e.g., [11, 24, 31, 33]).

TRE has shown benefits to glucose metabolism in other populations, including those with metabolic syndrome and diabetes [18, 31, 32], but not yet in the cancer population because it has not yet been tested [17]. We saw small improvements (reductions) in several glucose parameters, but our study was not powered to see statistically significant group-level effects. While cancer and chemotherapy can sometimes cause dysregulation of glucose parameters [10], and diabetes specifically is associated with cancer-related fatigue [21], not all cancer survivors have dysregulated glucose metabolism. Based on studies among people with diabetes [32], TRE may be particularly beneficial for glucose parameters for those with dysregulated glucose metabolism at baseline.

Actigraphy is a useful measure for circadian rest-activity rhythms in the cancer population, and parametric and non-parametric measures are complementary in describing the strength of the diurnal rhythm. For example, Liu *et al.* used wrist-worn actigraphs to show that people with breast cancer had disrupted rest-activity rhythms even before chemotherapy began, and that rhythms were less robust after chemotherapy [25]. Rhythms with less robusticity were associated with more fatigue [25]. Consistently, in this small study with considerable inter-individual variability, we saw that TRE led to trends towards higher cosinar and relative amplitudes and higher interdaily stability, though a larger study is necessary to achieve adequate power.

This study sets the stage for follow-on projects to further optimize TRE to address fatigue. The importance of the start time of the eating window is unknown, i.e., in relation to either daylight or an individual’s sleep patterns. Cancer-related fatigue is a multifaceted condition, and future research is warranted to explore who in particular will benefit from TRE in regard to cancer type, treatment type, clinical characteristics, or behavioral habits. Future research should also explore whether TRE can be combined with other interventions that entrain circadian rhythms, for example bright light therapy, for additive or synergistic effects.

This study has several strengths. Our population was diverse in regard to sex/gender, age, and race, increasing the generalizability to other cancer survivors. Also, we completed all study activities completely remotely, facilitating the ability to implement and disseminate a TRE program in clinical practices in the future. Our study was randomized with known potential confounding factors distributed fairly equally, and therefore any differences between groups can be attributed to TRE. Further, we had an active control condition to help control for time, attention, expectation of benefit, and potential improvements in the *quality* of diet, which may help discern the specific effects of the TRE eating pattern.

However, this study was not without limitations. Our drop-out rate was high at the beginning of our study, especially before randomization; but we were able to reduce dropout by increasing engagement later in the study. In addition, we recruited a heterogeneous population in regard to cancer type and treatment history; while that may increase generalizability, it may reduce our ability to see benefits if TRE is only effective for a subset of the eligible participants.

## Conclusions

There has been a recent explosion of exploration into “chrononutrition,” “chronochemotherapy,” and other “chronomedicine” approaches to understand how we can manipulate and harness circadian processes to prevent and treat chronic illnesses [9, 16]. Herein, we provide promising results from a randomized controlled trial that TRE may be able to alleviate persistent cancer-related fatigue. Given the appeal of TRE in regard to accessibility, low cost, low risk, and potential benefits, the results herein support follow-on studies to continue to evaluate TRE to address fatigue and other supportive care outcomes, as well as understand the underlying mediators so that we can tailor behavioral interventions and facilitate survivors’ recovery to life “before cancer.”

## Supplementary Material

Supplement 1

## Figures and Tables

**Table 1.** Demographics, clinical characteristics, and lifestyle habits^a^

	Consented (n=50)	Randomized (n=30)	Control (n=15)	Time-restricted eating (n=15)
**Characteristic**				
**Age (years)**	58.2±13.7	54.1±14.7^b^	53.7±16.8	54.6±12.7
**Sex**				
Female	36 (72.0%)	23 (76.7%)	10 (66.7%)	13 (86.7%)
Male	14 (28.0%)	7 (23.3%)	5 (33.3%)	2 (13.3%)
**Race**				
Black/African American	24 (48.0%)	16 (53.3%)	6 (40.0%)	10 (66.7%)
White	24 (48.0%)	13 (43.3%)	8 (53.3%)	5 (33.3%)
Other	1 (2.0%)	0	0	0
Mixed race	1 (2.0%)	1 (3.3%)	1 (6.7%)	0
**Ethnicity**				
Hispanic	2 (4.0%)	2 (6.7%)	0	2 (13.3%)
Non-Hispanic	48 (96.0%)	28 (93.3%)	15 (100%)	13 (86.7%)
**Living situation** ^b^				
Married or in long-term committed relationship	23 (46.0%)	16 (53.3%)	6 (40.0%)	10 (66.7%)
Single, divorced, or widowed	13 (26.0%)	10 (33.3%)	7 (46.7%)	3 (20.0%)
**Employment status**				
Employed full-time (≥35 hours/week)	16 (32.0%)	12 (40.0%)	8 (53.3%)	4 (26.7%)
Employed part-time (<35 hours/week)	5 (10.0%)	4 (13.3%)	3 (20.0%)	1 (6.7%)
Employed, unknown hours/week	1 (2.0%)	1 (3.3%)	0	1 (6.7%)
Home Maker	2 (4.0%)	2 (6.7%)	1 (6.7%)	1 (6.7%)
Retired	8 (16.0%)	4 (13.3%)	0	4 (26.7%)
Unemployed/on leave	4 (8.0%)	3 (10%)	1 (6.7%)	2 (13.3%)
**Education**				
High school	7 (14.0%)	3 (10.0%)^b^	1 (6.7%)	2 (13.3%)
Some college	7 (14.0%)	5 (16.7%)	3 (20.0%)	2 (13.3%)
4-Year college degree	8 (16.0%)	5 (16.7%)	4 (26.7%)	1 (6.7%)
Graduate school	13 (26.0%)	13 (43.4%)	5 (33.3%)	8 (53.3%)
**Body mass index (kg/m** ^**2**^ **)**	30.4±5.9	29.8±5.8	28.7±4.3	30.9±7.0
**Exercise habits**				
Meets WHO^c^ recommendations	24/36 (66.7%)	22 (73.3%)	11 (73.3%)	11 (73.3%)
Does not meet WHO recommendations	12/36 (33.3%)	8 (26.7%)	4 (26.7%)	4 (26.7%)
**Cancer type**				
Leukemia	8 (16.0%)	6 (20.0%)	4 (36.7%)	2 (13.3%)
Lymphoma	9 (18.0%)	4 (13.3%)	1 (6.7%)	3 (20.0%)
Multiple myeloma	27 (54.0%)	16 (53.3%)	8 (53.3%)	8 (53.3%)
Solid tumor	6 (12.0%)	4 (13.3%)	2 (13.3%)	2 (13.3%)
**Treatment for cancer**				
Surgery	8 (16.0%)	6 (20.0%)	4 (26.7%)	2 (13.3%)
Chemotherapy	47 (94.0%)	28 (93.3%)	14 (93.3%)	14 (93.3%)
Radiation	8 (16.0%)	7 (23.3%)	4 (26.7%)	3 (20.0%)
Stem cell transplant	20 (40.0%)	11 (36.7%)	5 (33.3%)	6 (40.0%)
CAR-T cell therapy	11 (22.0%)	5 (16.7%)	3 (20.0%)	2 (13.3%)
**Charlson Comorbidity Index**	2.9±1.7	2.6±1.3	2.7±1.0	2.5±1.5

a Some missing data exist.

b Differences were observed between those randomized and not randomized (*t*-test or χ^2^ likelihood ratio test, *p*<0.05). There were no statistically significant differences between time-restricted eating and control groups.

c World Health Organization, a combination of moderate- and vigorous-intensity physical activity achieving at least 600 metabolic equivalent-minutes per week, as measuring using a self-administered Global Physical Activity Questionnaire [2].

**Table 2.** Fatigue over time by group

Fatigue measure	Directionality	Group	Baseline (Mean ±SD)	Week 6 (Mean ±SD)	Effect size of TRE vs. Control* at Week 6	Week 12 (Mean ±SD)	Effect size of TRE vs. Control at Week 12
Control *n*			15	13		14	
TRE *n*			15	12		11	
FACIT-F Physical well-being	Higher is better	Control	22.0 ± 4.6	23.1 ± 2.8	0.33	22.9 ± 3.7^a,b**^	0.45
		TRE	18.5 ± 6.1	20.9 ± 4.1		21.7 ± 5.1	
FACIT-F Social well-being	Higher is better	Control	22.9 ± 4.2	23.0 ± 3.6	−0.19	22.8 ± 5.1	−0.17
		TRE	22.5 ± 4.8	21.1 ± 5.4		21.9 ± 4.0	
FACIT-F Emotional well-being	Higher is better	Control	19.0 ± 3.1	20.5 ± 2.3	−0.06	20.5 ± 2.8^a^	0.44
		TRE	18.9 ± 3.5	18.9 ± 3.4		20.8 ± 2.6	
FACIT-F Functional well-being	Higher is better	Control	18.8 ± 5.0	19.6 ± 4.7	0.03	20.2 ± 6.7	−0.33
		TRE	18.5 ± 5.8	18.8 ± 5.0		18.7 ± 5.4	
FACIT-F Fatigue-specific well-being	Higher is better	Control	36.7 ± 9.2	37.8 ± 8.6	0.25	37.2 ± 9.1^a,c^	0.70
		TRE	33.8 ± 11.4	35.2 ± 10.9		38.7 ± 9.2	
FACIT-F Total score	Higher is better	Control	120.1 ± 21.3	123.9 ± 14.2	0.14	123.7 ± 22.6	0.30
		TRE	112.1 ± 26.8	114.9 ± 20.4		121.8 ± 21.6	
Brief Fatigue Inventory: Global score	Lower is better	Control	3.4 ± 1.8	2.6 ± 1.8	0.08	3.5 ± 2.8^c^	−0.53
		TRE	3.9 ± 2.7	3.3 ± 2.5		2.8 ± 2.3	
Brief Fatigue Inventory: Usual fatigue	Lower is better	Control	4.3 ± 2.4	3.4 ± 2.5	0.27	4.1 ± 2.6^a^	−0.45
		TRE	4.4 ± 2.9	3.8 ± 2.6		2.8 ± 3.1	
Brief Fatigue Inventory: Fatigue at its worst	Lower is better	Control	5.1 ± 2.7	3.2 ± 2.0*	0.71	2.8 ± 2.4^a**^	−0.37
		TRE	5.1 ± 3.1	4.8 ± 3.2		2.6 ± 3.4	
Brief Fatigue Inventory: Interference of fatigue with enjoyment of life	Lower is better	Control	3.1 ± 3.0	2.3 ± 2.2	0.07	2.9 ± 2.4^a^	−0.43
		TRE	3.6 ± 3.6	3.4 ± 3.4		2.5 ± 2.8	

* Effect size is calculated from the change scores from baseline to week 6 or baseline to week 12.

a *p*<0.15 over time in a mixed model

b *p*<0.15 by group in a mixed model

c *p*<0.15 for group×time in a mixed model

** *p*<0.05

**Table 3.** Results of mixed model analyses evaluating the effects of TRE vs. control on rest-activity parameters (*n*=67 observations for non-parametric measures and *n*=65 for parametric measures).

Parameter	Definition	Interpretation	Effect of group (TRE vs. Control, Estimate ±SE)	*p*-value	Effect of time (Estimate ±SE)	*p*-value	Effect of group (TRE vs. control)*Time(Estimate ± SE)	*p*-value
Interdaily stability	The degree of regularity in the rest-activity pattern (range 0–1)	Higher is better	−0.016±0.020	0.421	0.002±0.003	0.5386	0.003±0.003	0.194
Intradaily variability	The degree of fragmentation of rest-activity periods (range 0–2)	Lower is better	−0.028±0.042	0.505	0.005±0.006	0.3801	−0.001±0.006	0.857
Least 5 average	The average activity levelfor thesequence ofthe least fi veactive hours(fromaveraged 24-hour periodsof an overlayof all days)	Lower is better	−114.5±132.1	0.394	−7.45±14.8	0.6168	−14.5±14.8	0.334
Least 5-Start hour	The onset of the “Least 5” sequence [range (midnight) to 24 (midnight the following day)]*	Descriptive	−0.439±0.221	0.057	−0.004±0.028	0.8768	0.035±0.028	0.216
Most 10 average	The average activity level for the sequence of the most 10 active hours (from averaged 24-hour periods of an overlay of all days)	Higher is better	158.6±716.3	0.827	20.4±105.6	0.8478	68.4±105.6	0.521
Most 10-Start hour	The temporal onset of the “Most 10” sequence [range 0 (midnight) to 24 (midnight the following day)]	Descriptive	−0.398±0.378	0.303	−0.030±0.037	0.4279	−0.004±0.037	0.903
Relative amplitude	[(Most 10)-(Least 5)]/[(Most 10)+(Least 5)] The range is 0–1.	Higher is better	0.023±0.018	0.217	0.000±0.002	0.9716	0.003±0.002	0.239
Fitted cosine Peak	Where in the 24-hour period the peak is [range 0 (midnight) – 1 (midnight the next day)]	Descriptive	−0.013±0.012	0.285	0.001±0.001	0.464	0.001±0.001	0.432
Fitted cosine Amplitude	Amplitude of fitted cosine curve (positive number, no defined range)	Higher is better	0.187±1.849	0.920	0.173±0.263	0.5164	0.325±0.263	0.226
Fitted cosine MESOR	Midline estimating statistic of rhythm from the cosine curve (positive number, no defined range)	Neither	−0.004±1.962	0.998	0.230±0.236	0.3366	0.174±0.236	0.467

* For individuals in which the L5 started before midnight, the time was converted to the hour(s) before midnight, for example “−1” for 11pm.
