# Supplementary material for: Time-restricted eating to address persistent cancer-related fatigue among cancer survivors: A randomized controlled trial
Source: Res Sq. 2024 Dec 25:rs.3.rs-5530166. Preprint. [Version 1] doi: 10.21203/rs.3.rs-5530166/v1 (PMC11703331; doi:10.21203/rs.3.rs-5530166/v1)
Supplement: Supplement 1 [file NIHPPRS5530166V1-supplement-1.pdf]

Supplementary Files

This is a list of supplementary files associated with this preprint. Click to download.

- suppl.docx
